# Carbon Acidity in Enzyme Active Sites

**DOI:** 10.3389/fbioe.2019.00025

**Published:** 2019-02-19

**Authors:** Michael D. Toney

**Affiliations:** Department of Chemistry, University of California, Davis, Davis, CA, United States

**Keywords:** carbon acid, enzyme, pyridoxal phosphate, PKA, general acid/base catalysis, marcus theory, carbanion stability

## Abstract

The pK_a_ values for substrates acting as carbon acids (i.e., C-H deprotonation reactions) in several enzyme active sites are presented. The information needed to calculate them includes the pK_a_ of the active site acid/base catalyst and the equilibrium constant for the deprotonation step. Carbon acidity is obtained from the relation pK_eq_ = p${\text{K}}_{\text{a}}^{\text{r}}$–p${\text{K}}_{\text{a}}^{\text{p}}$ = ΔpK_a_ for a proton transfer reaction. Five enzymatic free energy profiles (FEPs) were calculated to obtain the equilibrium constants for proton transfer from carbon in the active site, and six additional proton transfer equilibrium constants were extracted from data available in the literature, allowing substrate C-H pK_a_s to be calculated for 11 enzymes. Active site-bound substrate C-H pK_a_ values range from 5.6 for ketosteroid isomerase to 16 for proline racemase. Compared to values in water, enzymes lower substrate C-H pK_a_s by up to 23 units, corresponding to 31 kcal/mol of carbanion stabilization energy. Calculation of Marcus intrinsic barriers (Δ${\text{G}}_{0}^{\text{‡}}$) for pairs of non-enzymatic/enzymatic reactions shows significant reductions in Δ${\text{G}}_{0}^{\text{‡}}$ for cofactor-independent enzymes, while pyridoxal phosphate dependent enzymes appear to increase Δ${\text{G}}_{0}^{\text{‡}}$ to a small extent as a consequence of carbanion resonance stabilization. The large increases in carbon acidity found here are central to the large rate enhancements observed in enzymes that catalyze carbon deprotonation.

Mechanistic enzymologists have made great strides over the past decades in deciphering the fundamental principles of enzyme catalysis. Nevertheless, a quantitative accounting of the contributions to rate enhancement has not yet been achieved (Machleder et al., 2010; Wolfenden, 2011; Herschlag and Natarajan, 2013; Richard, 2013; Warshel and Bora, 2016). One of the most fundamental catalytic mechanisms available to enzymes is general acid/base catalysis by amino acid side chains in active sites (Jencks, 1987; Richard, 1998; Frey and Hegeman, 2007). Deprotonation of carbon acids (C-H bonds of substrates) is an especially important and difficult reaction requiring base catalysis (Richard and Amyes, 2001; Richard, 2012). Enzymologists have measured the pK_a_ values of many active site catalytic residues through pH-rate profiles (Cook and Cleland, 2007), and these can frequently be assigned to specific residues in combination with additional information, but substrate C-H pK_a_ values have remained elusive.

Many enzymatic reactions involve deprotonation of carbon as a central step in the catalytic mechanism, yet there are no examples in the literature where the pK_a_ of a substrate C-H has been established experimentally. The closest example known to this author is that of uridine monophosphate bound to orotidine monophosphate decarboxylase (Amyes et al., 2008). In that work, the authors estimated the pK_a_ of the product (≤22) by isotope exchange kinetics, which generates via deprotonation the same vinyl carbanion resulting from decarboxylation. It is generally appreciated that enzymes must substantially lower pK_a_s of carbon acids at active sites to achieve observed rate enhancements (Gerlt et al., 1991; Gerlt and Gassman, 1992, 1993a,b; Richard et al., 2014). A full understanding of the thermodynamics, including the pK_a_ values for both the general acid/base catalyst and the substrate C-H bond is needed to account quantitatively for enzyme catalysis.

Here, a recently introduced method for free energy profile (FEP) determination (Toney, 2013) is applied to five enzymes, employing experimental data reported in the literature. The FEPs allow calculation of proton transfer equilibrium constants in active sites. Additionally, literature FEPs and spectroscopic information are used to calculate proton transfer equilibrium constants for six additional enzymes. Combining proton transfer equilibrium constants with pK_a_s for catalytic active site residues allows one to solve for active site-bound substrate C-H pK_a_s, which are in the range ~6 to ~16 for the enzymes discussed here.

## Methods

Here, FEP determination involves optimizing the agreement between several calculated and observed experimental measurements simultaneously. The freely available biochemical simulation and analysis software COPASI was used for all optimizations (Hoops et al., 2006; Mendes et al., 2009). The procedure used here does not involve time-consuming numerical integration of differential rate equations. Instead, the adjustable parameters (rate constants) are altered by the chosen algorithm and the new parameters are used to calculated a new value of the target function (see below) (Toney, 2013). This is much less computationally demanding than fitting to primary kinetic data via numerical integration, allowing essentially exhaustive exploration of parameter space. Global optimization algorithms fall into four main categories: random, deterministic, stochastic (e.g., simulated annealing), and heuristic (e.g., genetic algorithms, swarm algorithms) (Moles et al., 2003). COPASI implements examples of all these categories. The COPASI input files used here for FEP determinations are included in the Supplementary Material.

A critical step to defining enzymatic FEPs by global optimization is the specification of the target function to be minimized. A sum-of-squared absolute values of residuals between calculated and experimental values, divided by the experimental value, was used. Equation (1) shows the target function used for alanine racemase.

(1) $$\begin{array}{l} SSR={\left|\frac{k_{L}^{calc}-k_{L}^{expt}}{k_{L}^{expt}}\right|}^{2}+{\left|\frac{k_{D}^{calc}-k_{D}^{expt}}{k_{D}^{expt}}\right|}^{2}+{\left|\frac{K_{L}^{calc}-K_{L}^{expt}}{K_{L}^{expt}}\right|}^{2}\\ \;+{\left|\frac{K_{D}^{calc}-K_{D}^{expt}}{K_{D}^{expt}}\right|}^{2}+{\left|\frac{Visc^{calc}-Visc^{expt}}{Visc^{expt}}\right|}^{2}+\text{ ​​}\;\text{​​ }etc.\text{ ​​}\; \end{array}$$

Here, k_L_ is k_cat_ for the L → D direction, K_L_ is K_M_ for the L → D direction, “Visc” is the effect of viscosity on relative k_cat_/K_M_ values, etc. Central to the procedure, random initial values for all parameters were assigned automatically by COPASI at the beginning of each individual optimization run. The use of the mean normalized difference between calculated and observed values weights the different experimental measurements equally. This is essentially a sum of chi-squared statistics (Greenwood and Nikulin, 1996). It is analogous to the commonly used relative weighting scheme in non-linear regression (Motulsky and Christopoulos, 2004).

Microscopic rate constants and intrinsic kinetic isotope effects (KIEs) (where applicable) were adjustable parameters. For bimolecular rate constants, the lower bound was k_cat_/K_M_ for the respective direction, and the upper bound was 10^9^ M^−1^s^−1^ (diffusion limit). For unimolecular constants, the lower bound was k_cat_ for the respective direction, and the upper bound was 10^12^ s^−1^ (vibrational limit). The values of intrinsic deuterium KIEs were limited to the semi-classical range of 1–6. The application of these limits is important for restricting the parameter space searched to a productive one.

The search of parameter space was performed in two phases. First, a broad search over the rate constant limits given above was performed using the “genetic algorithm” in COPASI. Second, a focused search was performed to define well the sum of squared residuals (SSR) surface at the lower SSR values: narrower limits on each parameter (corresponding to a 50-fold increase in SSR from the lowest values obtained in the first search) were set. The latter employed the “particle swarm” algorithm in COPASI. A complete search was comprised of 10^5^-10^6^ *independent* calculations. Each calculation started with random initial values for the parameters, within the specified limits. This was automated using the “parameter scan” task in COPASI.

## Results and Discussion

The calculation of C-H pK_a_ values in enzymes active sites reported here employs the relationship between reactant and product pK_a_ values for a simple proton transfer reaction:

(2) $$pK_{eq}=pK_{a}^{reactant}-\text{ ​​}\;\text{​​ }pK_{a}^{product}$$

The equilibrium constant (pK_eq_) for the proton transfer between a carbon acid and an acid/base catalyst in the active site and the pK_a_ of the product must be known to solve for the reactant (C-H) pK_a_. For general base catalysis by an active site amino acid side chain, the pK_a_ of the product is the pK_a_ of the protonated form of the side chain in the enzyme-substrate complex, which is readily obtained from k_cat_ vs. pH profiles: k_cat_/K_M_ vs. pH profiles provide pK_a_ values for free enzyme and free substrate, while k_cat_ vs. pH profile provides pK_a_ values for enzyme-substrate complexes. The latter are relevant to the calculation of substrate C-H pK_a_s in active sites and are used here.

The equilibrium constant is generally more difficult to obtain. The simplest method, but applicable only to select classes of enzymes, is to use spectroscopic information (e.g., absorbance and extinction coefficient) that is specific to the carbanionic intermediate to calculate the equilibrium constant. Pyridoxal phosphate (PLP) dependent enzymes constitute an especially favorable case since the highly resonance stabilized carbanionic “quinonoid” intermediate has long wavelength absorption bands (~500 nm) with high a extinction coefficient (~40,000 M^−1^cm^−1^) that allow it to be readily identified and quantified (Metzler et al., 1988; Mozzarelli et al., 2000).

**Scheme 1:**
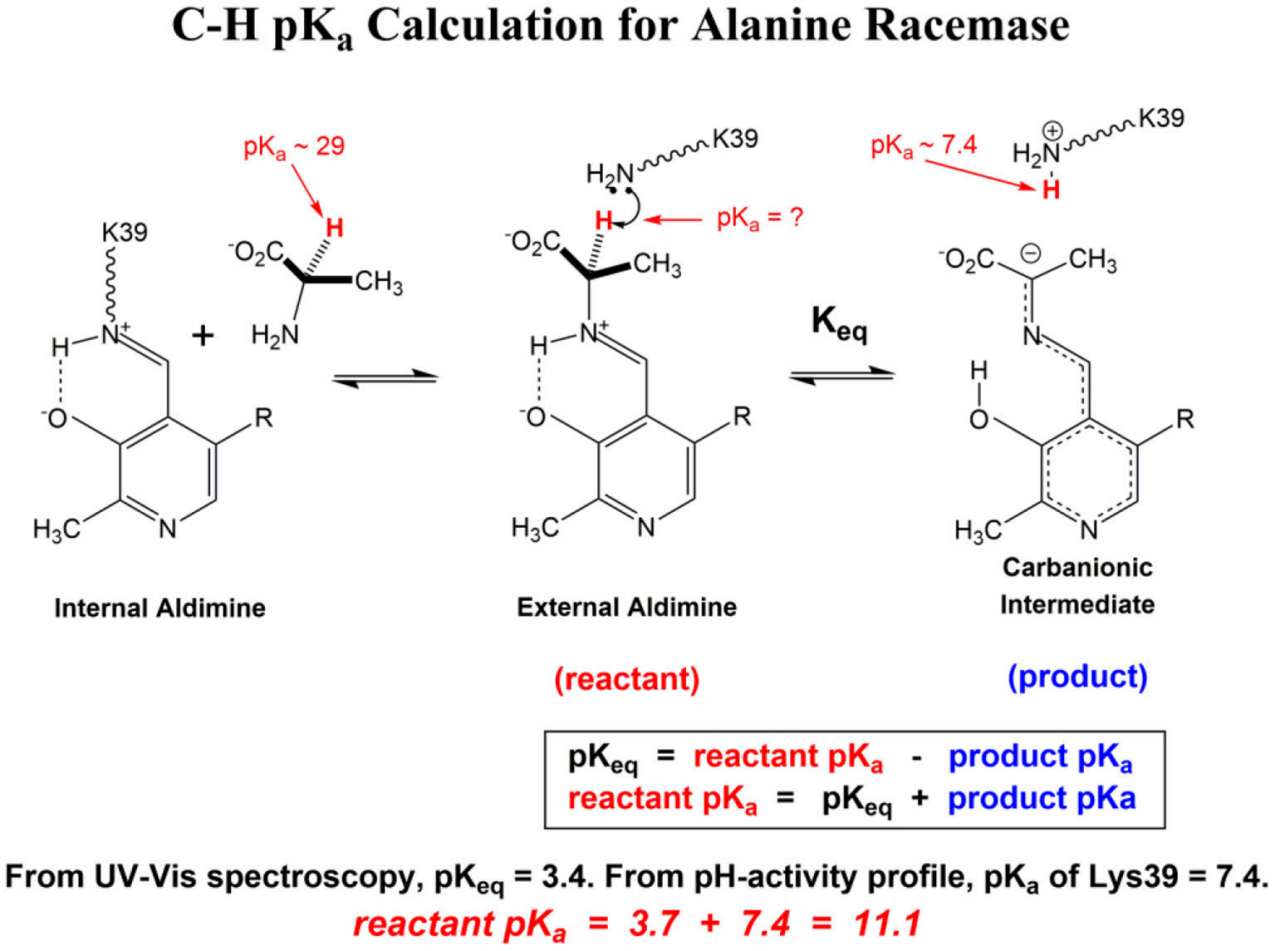
C-H pK calculation for Alanine Racemase.

Scheme 1 Illustrates the calculation of the C-H pK_a_ for alanine bound to alanine racemase (AR) based on spectroscopic information. From published pH profiles for k_cat_, one can deduce a pK_a_ of 7.4 for Lys39 in the substrate-bound active site (Sun and Toney, 1999). From UV-vis spectroscopy of AR saturated with alanine, one can calculate pK_eq_ = 3.4 for proton transfer based on the 500 nm absorption band of the carbanionic quinonoid intermediate (Toney, 2013). Thus, the external aldimine C-H pK_a_ = 3.7 + 7.4 = 11.1. The C-H pK_a_ values of 12 active site-bound substrates are presented in Table 1, along with the corresponding pK_a_s in water, the difference in pK_a_ for free vs. active site bound substrates, and pK_a_s calculated via QM/MM simulations where available.

**Table 1:** Carbon Acid (Substrate C-H) pK_a_ values.

**Enzyme (substrate)**	**Water pK_**a**_ (experimental)^a^**	**Active site pK_**a**_ (experimental)^b^**	**Active site pK_**a**_ (QM/MM)**	**ΔpK_**a**_^c^**
**COFACTOR INDEPENDENT ENZYMES**				
Ketosteroid isomerase (Δ-3-keto steroid)	13	5.6	5.6	7.4
Triosephosphate isomerase (GAP)	17	*9–12*	11	5–8
Triosephosphate isomerase (DHAP)	18	*10–14*	14–20	4–8
Proline racemase (Proline)	29	16	16	13
Mandelate Racemase (Mandelate)	30	*9–15*	17	15–21
Fumarase (Malate)	30	*9–13*	–	17–21
**PYRIDOXAL PHOSPHATE DEPENDENT ENZYMES**				
Tryptophan synthase (Tryptophan)	29	8	–	21
Tryptophan indole-lyase (Tryptophan)	29	6	–	23
Tyrosine phenol-lyase (Phenylalanine)	29	6	–	23
Alanine Racemase (Alanine)	29	11	12	18
Aspartate aminotransferase (Aspartate)	29	~7	–	~22
Dialkylglycine decarboxylase (Alanine)	29	8	–	21

a The references for C-H pK_a_ values for substrates are given in Supplementary Material.

b Experimental active site C-H pK_a_ ranges are determined from the lower limit for the carbanion reprotonation rate constant obtained from the FEP and an assumed upper limit of 10^12^ s^-1^. Values that are not well-defined are highlighted in italics.

C *The difference in C-H pK_a_ between the substrate in water and in the enzyme active site*.

A general method for evaluating the proton transfer equilibrium constant is to calculate a FEP for the complete enzyme catalyzed reaction, then take the ratio of the deprotonation to reprotonation rate constants as the proton transfer equilibrium constant. Historically, the determination of FEPs for enzymes was a laborious process requiring a variety of enzyme-specific experiments, generally including pre-steady-state kinetic measurements. Recently, this author showed that enzymatic FEPs are readily obtained by combining the information obtained from a variety of commonly employed enzyme kinetic experiments (Toney, 2013). The types of information combined include, for example, k_cat_ and K_M_, KIEs, viscosity effects, washout vs. turnover ratios, etc. The key is that these different experimental measurements provide information on various components of the reaction sequence constituting the enzymatic mechanism. The experimental data are combined in a target function for global optimization, in which the individual rate constants for the enzymatic mechanism are optimized via a minimization algorithm (e.g., genetic algorithm, particle swarm, Hooke, and Jeeves, etc.) to achieve best-fit agreement between calculated and experimental observations.

This method was employed here to calculate five new enzymatic FEPs. All FEPs were calculated with COPASI (Hoops et al., 2006; Mendes et al., 2009). The COPASI input files used here are provided separately as Supplementary Material. The general mechanism used for the analysis of all the enzymes considered here is:

(3) $$E+S\;\overset{k1}{\underset{k2}{⇌}}\;ES\;\overset{k3}{\underset{k4}{⇌}}\;EI\;\overset{k5}{\underset{k6}{⇌}}\;EP\;\overset{k7}{\underset{k8}{⇌}}\;E+P$$

### Ketosteroid Isomerase (3-Oxo-Δ5-Steroid Isomerase) FEP

Pollack et al. extensively studied the reaction catalyzed by ketosteroid isomerase (KSI). Their work resulted in a nearly complete FEP calculated from a variety of different experiments (Hawkinson et al., 1991). They were unable to define a precise value for the energy of the enolate (enol) intermediate, only a lower limit. Therefore, FEP calculations to define these values was undertaken using global optimization. The experimental data employed included k_cat_ and K_M_ values for the forward reaction, the partitioning ratio for the intermediate going backward to substrate vs. forward to product, KIEs, the equilibrium constant for the reaction, the product dissociation constant, and two rate constant ratios (k_1−_/k_2_, and k_−2_/k_3_). The details of FEP calculations are provided in Supplementary Material.

**Figure 1:**
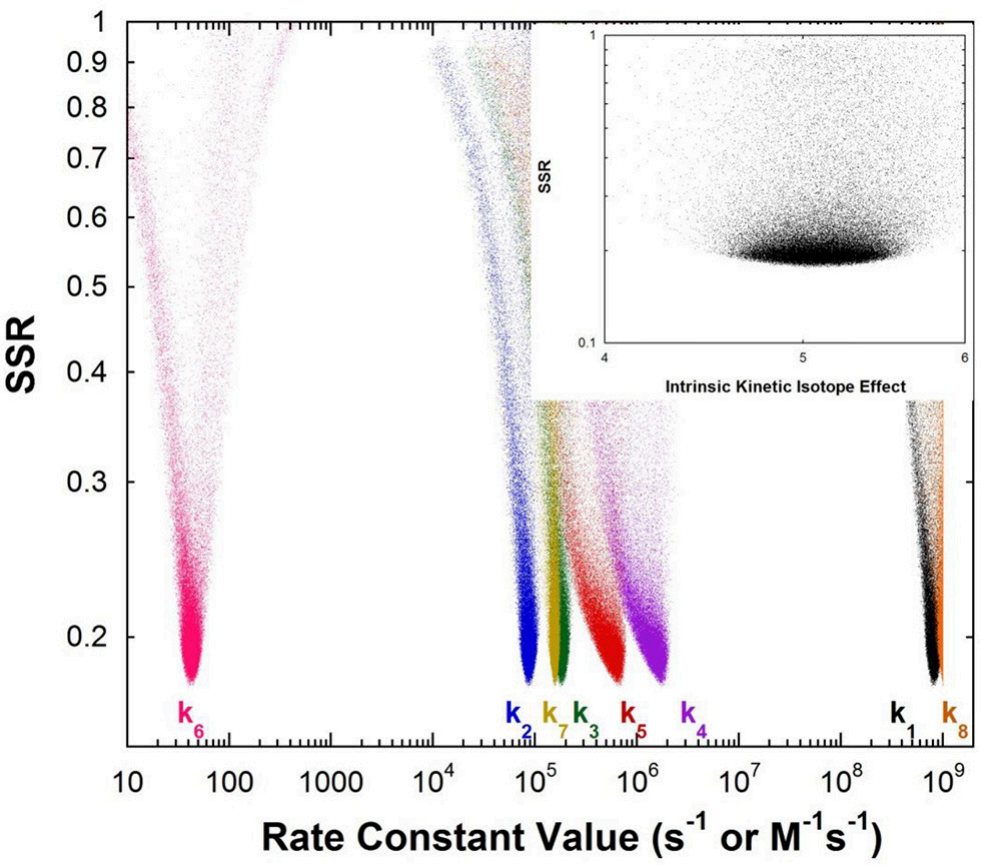
Results of global optimization with KSI. SSR, sum of squared residuals of the fit to the target function. Each point results from an independent optimization run. The figure was generated from ~100,000 independent runs, each starting from randomized sets of rate constants. The inset presents the intrinsic KIE resulting from global optimization.

Figure 1 shows the results of global optimization with KSI. The graph presents the sum-of-squared residuals (SSR), which is a measure of the goodness-of-fit to experimental data for a series of *independent* optimization runs, plotted against the fitted values of the rate constants. Each independent global optimization run results in a set of parameters (rate constants, KIE) with a common SSR value (identical y-axis value). The lower the value of the SSR, the better the optimized rate constants predict the experimental results.

Fundamentally, the graph shows how sensitive the goodness-of-fit is to the values of the fitted parameters (i.e., rate constants and intrinsic KIEs): rate constants with narrower SSR “peaks” at the bottom of the distributions are better defined. For example, the inset shows the SSR vs. the value of the intrinsic KIE on the deprotonation step. The lowest values of SSR occur at an intrinsic KIE of ~5, but the wide distribution shows that the fit to the experimental data is not very sensitive to the value of this parameter, and it is therefore not well-defined. On the other hand, k_7_ has a narrow SSR “peak” and its value is well-defined.

Each independent optimization run generates 8 rate constants and the intrinsic KIE (if KIE measurements are included). Crucially, ~100,000 independent optimization runs are presented in the graph, each starting from a set of random initial rate constant values (within reasonable chemical limits: >k_cat_/K_M_ and < 10^9^ M^−1^s^−1^ for second order rate constants, and >k_cat_ and < 10^12^ s^−1^ for first order rate constants), constituting an essentially exhaustive search of rate constant space. The randomization of the initial guesses for the rate constants combined with the user-specified global optimization termination conditions provide the distribution of values that allow the “SSR surface” to be defined (i.e., not all fits advance to the absolute SSR minimum).

The resulting rate constant values for KSI are: *k*_1_ = 8.3 × 10^8^ M^−1^s^−1^, *k*_2_ = 8.6 × 10^4^ s^−1^, *k*_3_ = 1.8 × 10^5^ s^−1^, *k*_4_ = 1.7 × 10^6^ s^−1^, *k*_5_ = 6.4 × 10^5^ s^−1^, *k*_6_ = 43 s^−1^, *k*_7_ = 1.5 × 10^5^ s^−1^, *k*_8_ = 1 × 10^9^ M^−1^s^−1^. *These agree well with those reported by Pollack et al*, and show that global optimization can additionally define the rate constants for carbanion reprotonation (*k*_4_ and *k*_5_) that were previously not well-defined. The equilibrium constant for proton transfer (K_eq_) calculated from *k*_3_ and *k*_4_ is 0.11. This is in agreement with the previously calculated value of 0.3 ± 0.2 (Hawkinson et al., 1994).

The value of pK_eq_ for the reaction of 5-androstene-3,17-dione is 0.96 while that for 4-androstene-3,17-dione is 4.2. These values correspond to ΔG_0_ for proton transfer of 1.3 and 5.7 kcal/mol. They can be combined with the pK_a_ (4.6) of the general acid/base catalyst in the active site (Asp38) to give calculated active site C-H pK_a_ values of 5.6 for 5-androstene-3,17-dione and 8.8 for 4-androstene-3,17-dione. The pK_a_ of 5-androstene-3,17-dione in solution is 12.7 (Pollack et al., 1989).

**Scheme 2:**
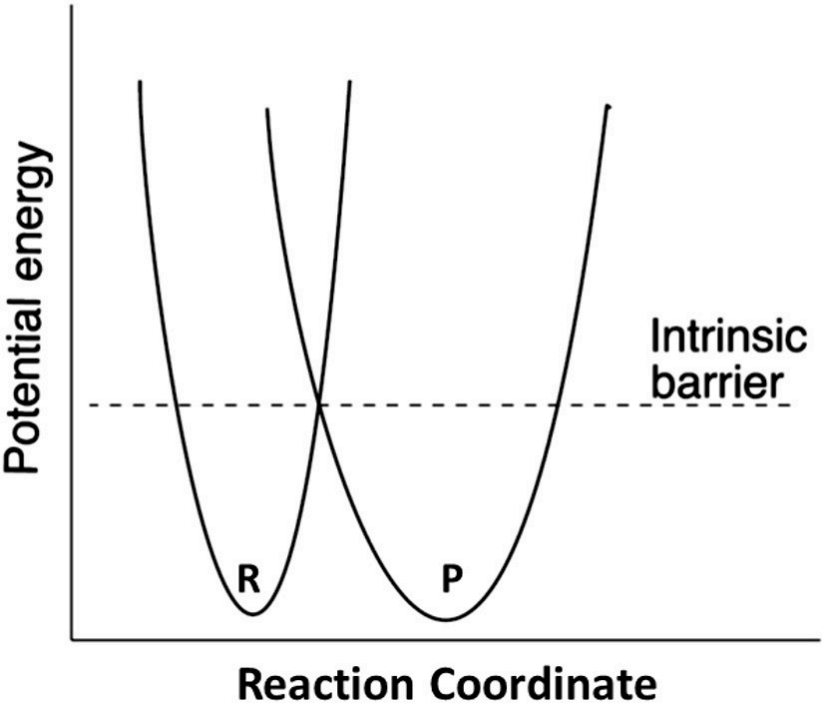
Definition of the Marcus intrinsic barrier.

Marcus theory for electron transfer has been extended to a variety of other reactions including proton transfers and enzymatic reactions (Silverman, 2000; Bearne and Spiteri, 2005). In its simplest form, the theory describes a reaction in terms of an intrinsic reaction barrier (Scheme 2), which is the barrier for the reaction when ΔG^0^ = 0 (kinetic component), and the difference in free energy between reactants and products (thermodynamic component). The theory has been extended to include work terms to describe the energy required to bring reactants together into the reactive ground state complex; this term is ignored here since enzymes form the reactive complex in a separate substrate binding step (i.e., substrate binding energy pays for the work required to form the reactive complex), and the calculations for the non-enzymatic reactions correct the measured second order rate constants for reactive complex formation by using an association constant of 0.017 M^−1^ estimated by Hine and commonly employed in the literature (Hine, 1971). The form of the Marcus equation used here is given in Equation (4), where ΔG^‡^ is the observed free energy of activation, ΔG^0^ is the energy difference between reactants and products, and Δ${\text{G}}_{0}^{\text{‡}}$ is the intrinsic reaction barrier.

(4) $$\Delta G^{‡}={\left(1+\Delta G^{o}/4\Delta G_{o}^{‡}\right)}^{2}\Delta G_{o}^{‡}$$

The Δ${\text{G}}_{0}^{\text{‡}}$ values for proton abstraction in ketosteroid isomerization were previously estimated to be 10 kcal/mol for the enzymatic reaction and 13 kcal/mol for the reaction in solution (Hawkinson et al., 1994). The rate constant values derived here from global optimization allow calculation of Δ${\text{G}}_{0}^{\text{‡}}$ = 9.5 kcal/mol for the enzymatic reaction (Table 2), in agreement with the previously calculated value.

**Table 2:** Marcus intrinsic barriers (kcal/mol)^a^.

**Enzyme**	**ΔG_**0**_ solution**	**ΔG_**0**_ active site**	**ΔΔG_**int**_**
Ketosteroid isomerase	13	9.5	−3.5
Proline racemase	10.5	5.1	−5.4
Tryptophan indole-lyase	10.4^b^	12.8	+2.4
Tyrosine phenol-lyase	10.4^b^	15.5	+5.1
Aspartate aminotransferase	10.4^b^	11.5	+1.1
Dialkylglycine decarboxylase	10.4^b^	11.3	+0.9

a *Intrinsic barriers to reaction based on Marcus theory, without including any work terms since the juxtaposed active site base catalytst and substrate react in a unimolecular step in the absence of bulk solvent. Calculation details in Supplementary Material*.

b *Calculated intrinsic barrier for Gly-pyridoxal aldimine in water*.

### Mandelate Racemase (MR) FEP

**Figure 2:**
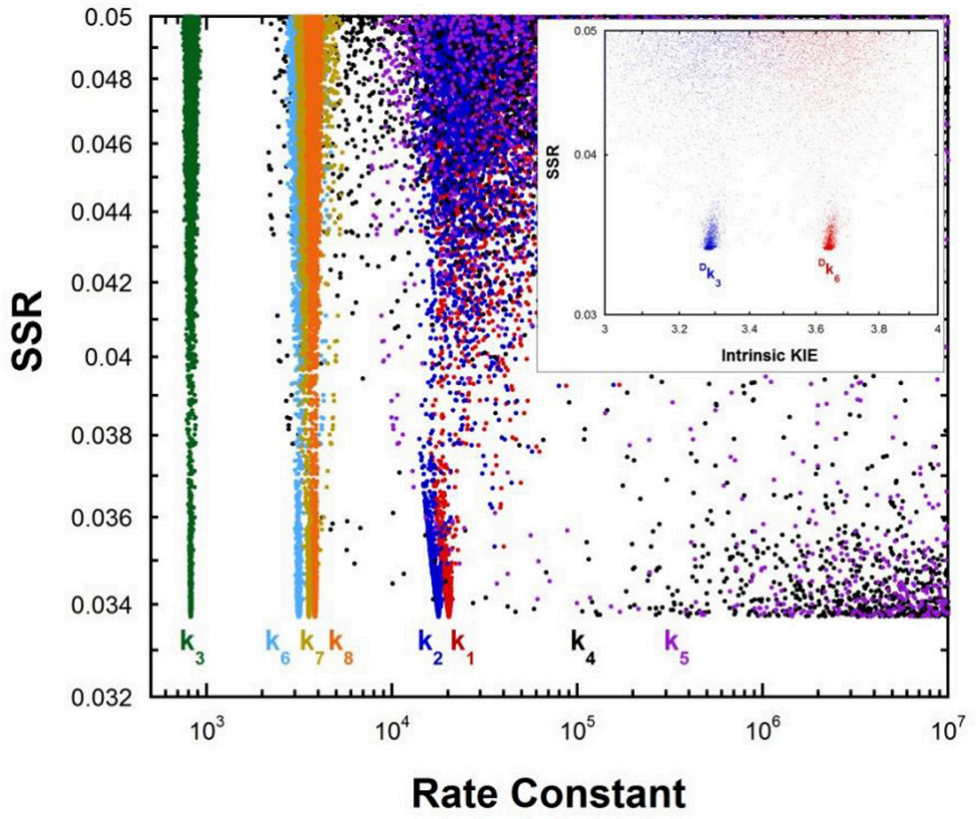
Results of global optimization with mandelate racemase. SSR, sum of squared residuals of the fit to the target function. Each point results from an independent optimization run. The figure was generated from ~40,000 independent runs, each starting from randomized sets of rate constants. The inset presents the intrinsic kinetic isotope effect resulting from global optimization. The fit to the target function is shows significant sensitivity to the values of *k*_4_ and *k*_5_ when they are < 10^6^ s^−1^. Therefore, these are the lower limits on the values of these rate constants.

Multiple KIE experiments provide good evidence for a carbanionic intermediate in mandelate racemase catalysis (Mitra et al., 1995), as does the partitioning of an alternative substrate between racemization and bromide elimination (Lin et al., 1988). This justifies the use of the mechanism in Equation (2) with MR. The FEP for MR was determined by combining k_cat_ and K_M_ for both directions of racemization with viscosity effects, KIEs, and intermediate partitioning (Whitman et al., 1985; Powers et al., 1991; St Maurice and Bearne, 2002). The global optimization results are presented in Figure 2. The pK_a_ of the active site acid/base catalyst is 6.4 (Kallarakal et al., 1995). From the results presented in Figure 2, the rate constant for deprotonation of (*S*)-mandelate in the active site (*k*_3_) is 800 s^−1^ while the reprotonation rate constant (*k*_4_) is in the range of 10^5^ and 10^12^ s^−1^. These values translate into a C-H pK_a_ range of 8.5–15.5, reported as 9–15 in Table 1. The reverse isomerization reaction occurs with a deprotonation rate constant (*k*_6_) of 3,300 s^−1^, while reprotonation (*k*_5_) is in the range of 10^6^-10^12^ s^−1^. These values correspond to C-H pK_a_ values of 9–15.

The C-H pK_a_ of mandelate (monoanion) in water is calculated by assuming the rate constants for deprotonation catalyzed by hydronium and hydroxide ions are equal at pH 7 and 25°C, given the pH independence of the reaction rate in this region (Bearne and Wolfenden, 1997). The rate constant for proton exchange at pH 7 and 25°C is 3 × 10^−13^ s^−1^, or 1.5 × 10^−13^ s^−1^ for the hydroxide catalyzed component. This value, divided by the concentration of hydroxide at pH 7, gives a rate constant of 1.5 × 10^−6^ M^−1^s^−1^, which can be used with the correlation between log(k_OH_) and carbon acid pK_a_ presented by Richard (Richard et al., 2001) to give an estimated solution C-H pK_a_ of 30 for mandelate. This value is in agreement with others estimated in the literature (Gerlt et al., 1991). It can be compared to the C-H pK_a_ of 22 for mandelic acid (Chiang et al., 1990).

### Fumarase FEP

**Figure 3:**
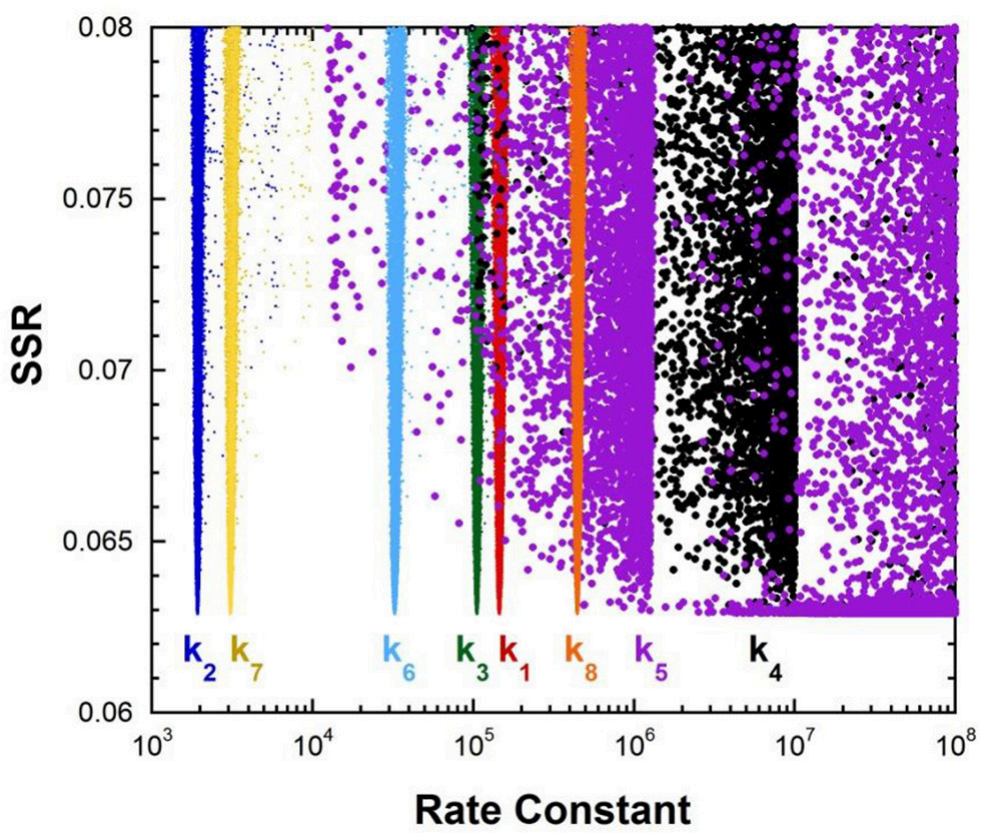
Results of global optimization with fumarase. SSR, sum of squared residuals of the fit to the target function. The figure was generated from ~40,000 independent runs, each starting from randomized sets of rate constants. The fit to the target function is shows significant sensitivity to the values of *k*_4_ and *k*_5_ when they are < 10^7^ s^−1^ and < 10^8^ s^−1^. Therefore, these are the lower limits on the values of these rate constants.

The fumarase FEP for pH 7 was calculated by global optimization, employing k_cat_ and K_M_ for both directions of the reaction, viscosity effects, the equilibrium constant, and rate constant ratios and commitments to catalysis determined by KIE analyses (Alberty and Peirce, 1957; Brant et al., 1963; Blanchard and Cleland, 1980; Sweet and Blanchard, 1990). The global optimization results are presented in Figure 3. The rate constant for deprotonation of malate in the active site (*k*_3_) is 1.1 × 10^5^ s^−1^, while the reprotonation rate constant (*k*_4_) is 10^7^-10^12^ s^−1^. These values allow calculation of a C-H pK_a_ of 8.5–13 in the active site (reported as 9–13 in Table 2), given the active site acid/base catalyst pK_a_ of 6.4 (Brant et al., 1963).

### Aspartate Aminotransferase FEP

**Figure 4:**
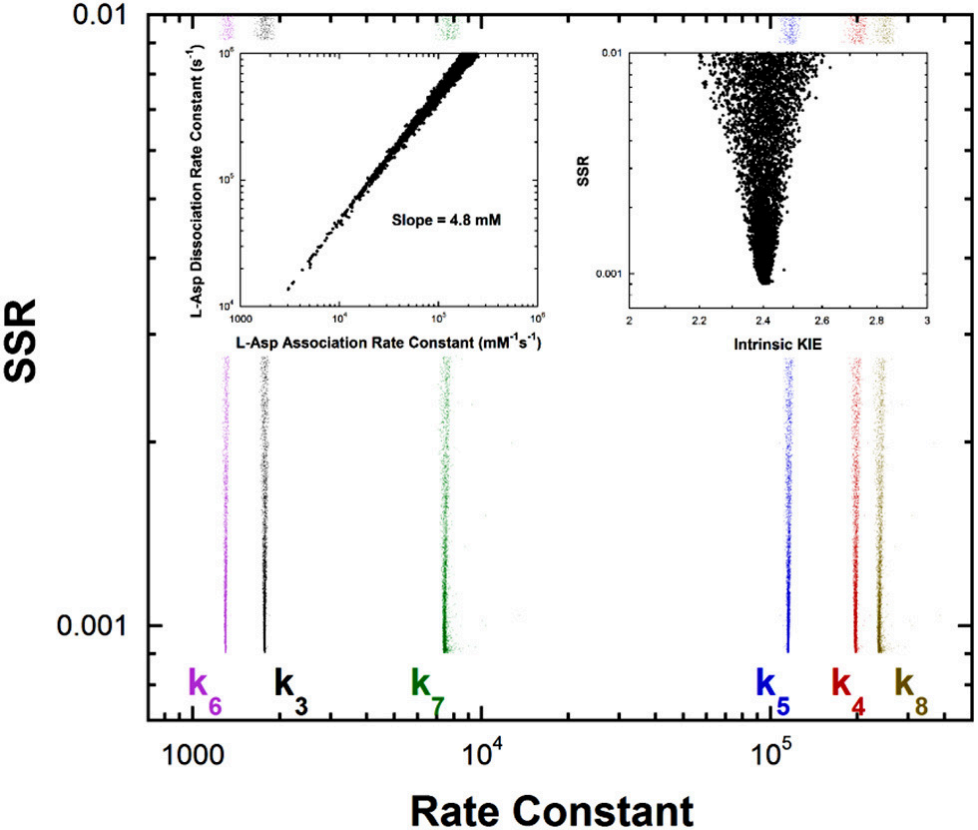
Results of global optimization for the L-asp/oxalacetate half-reaction of aspartate aminotransferase. SSR, sum of squared residuals of the fit to the target function. The figure was generated from ~40,000 independent runs, each starting from randomized sets of rate constants. The insets show that the correlation between the association and dissociation rate constants for L-Asp binding. The individual rate constants are not well-determined, but their ratio is over a broad range of values, indicating rapid equilibrium binding. The observed ratio of the rate constants is 4.8mM, which is the *K*_M_ value for L-Asp. The intrinsic KIE calculated in the global optimization is also presented as an inset.

Previous studies with aspartate aminotransferase defined rate constants for a mechanism in which the central 1,3-prototropic shift occurs as a concerted double proton transfer, avoiding the carbanionic quinonoid intermediate (Goldberg and Kirsch, 1996). A more recent study proved the existence of the quinonoid intermediate on the productive pathway (Hill et al., 2010). Therefore, a FEP including the quinonoid intermediate on the reaction pathway was determined by global optimization. Only the aspartate/oxalacetate half-reaction was analyzed. The experimental observations used in global optimization included pre-steady-state k_max_ and K_app_ from stopped-flow experiments, KIEs, viscosity dependence, intermediate partitioning (i.e., isotopic washout vs. turnover), the equilibrium constant, and the absorbance of the quinonoid intermediate, which were used in the previous study on the concerted mechanism. The results are presented in Figure 4.

The left inset to Figure 4 presents the correlation between the L-Asp association rate constant (*k*_1_) and the dissociation rate constant (*k*_2_). These composite rate constants include the steps leading from the free enzyme and free substrate up to and including external aldimine intermediate formation via transimination. The tight correlation between the rate constants, as well as the large value of *k*_2_ compared to *k*_3_, demonstrates that these steps are essentially at equilibrium with respect to the remainder of the half-reaction. The slope of the line (i.e., calculated equilibrium binding constant) is 4.8 mM, which is equal to the experimental K_app_ in stopped-flow analyses.

The remaining rate constants (*k*_3_-*k*_8_) in the mechanism are very well-defined by global analysis. The rate constant for external aldimine deprotonation is 1,800 s^−1^, while reprotonation occurs at 200,000 s^−1^. The value of pK_eq_ calculated from these rate constants is 2.0, close to the value of 2.3 calculated from UV-vis spectral data (Goldberg and Kirsch, 1996). The pK_a_ of the active site acid/base catalyst (Lys258) is taken here to be ~5.5, which is the value observed in the pH profile for k_cat_ with mutant enzymes (Y225F and K258C-EA) (Gloss and Kirsch, 1995). The value of k_cat_ for wild type and L-Asp shows pH dependence but the activity does not go to zero below the acidic pK_a_, making it unlikely that this ionization is that of Lys258, which is critical to catalysis (Toney and Kirsch, 1993). The combination of pK_eq_ = 2 and pK_a_ = ~5.5 gives an active site C-H pK_a_ for the external aldimine of 7.5, which is reported as ~7 in Table 1 due to the uncertainty in the pK_a_ of Lys258.

The FEP also allows calculation of the C-H pK_a_ for the C4′-H bond of the oxaloacetate ketimine intermediate. The pK_eq_ for C4′ deprotonation is calculated from the deprotonation rate constant of 1,300 s^−1^ and the reprotonation rate constant of 115,000 s^−1^ to be 2.0. Combined with the pK_a_ of ~5.5 for Lys258 this give a C4′-H pK_a_ of ~7.5 in the active site.

For enzymatic deprotonation of the external aldimine intermediate, ΔG^‡^ = 12.9 kcal/mol and ΔG_0_ = 2.7 kcal/mol for proton transfer. These values give Δ${\text{G}}_{0}^{\text{‡}}$ = 11.5 kcal/mol (Table 2).

### Dialkylglycine Decarboxylase FEP

**Figure 5:**
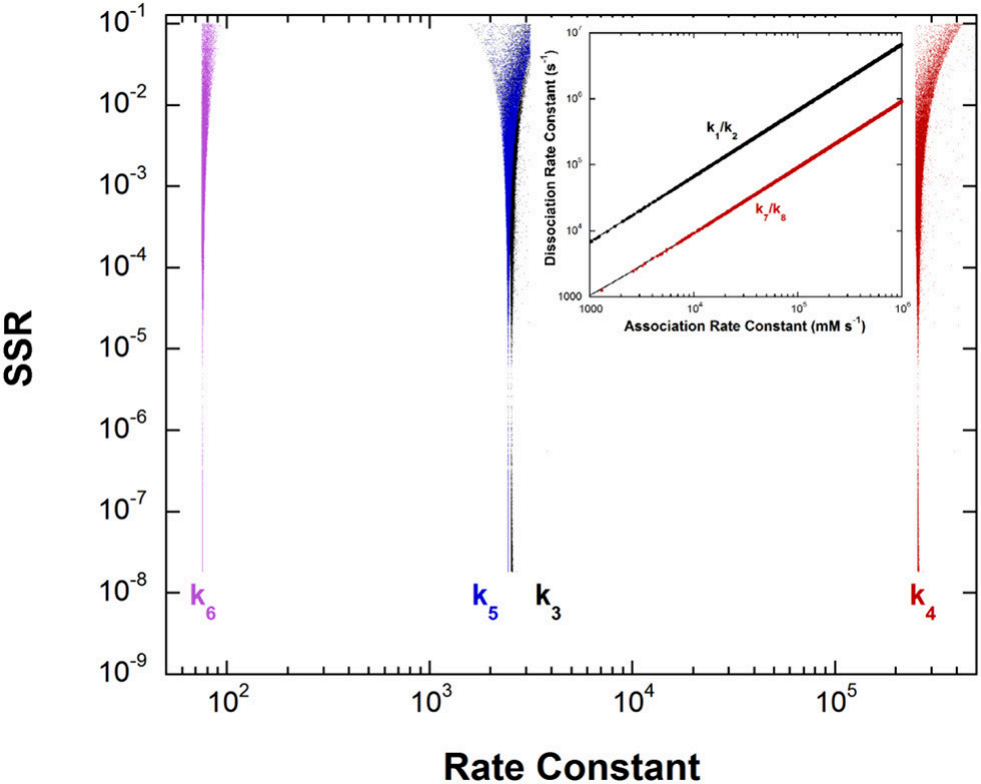
Results of global optimization for the L-Ala/pyruvate half-reaction of dialkylglycine decarboxylase. SSR, sum of squared residuals of the fit to the target function. The figure was generated from ~40,000 independent runs, each starting from randomized sets of rate constants. The inset shows the ratios of the association and dissociation rate constants for both L-Ala and pyruvate binding. The linear correlation over a very large range of values indicates rapid equilibrium binding of both.

This unusual PLP enzyme catalyzes the oxidative decarboxylation of 2,2-dialkylglycines in the first half-reaction of a ping-pong mechanism and the transamination of pyruvate to L-alanine in the second (Toney et al., 1995). The L-alanine transamination half-reaction was analyzed by global optimization. The experimental data included k_max_, K_app_, and rate constant ratios from stopped-flow experiments and intermediate partitioning (washout vs. turnover) (Zhou et al., 2001). The results are presented in Figure 5.

The inset shows that, as with aspartate aminotransferase, the formation of the external aldimine intermediate is at equilibrium (*k*_1_/*k*_2_). Additionally, the hydrolysis of the pyruvate ketimine intermediate and pyruvate dissociation (*k*_7_/*k*_8_) is also at equilibrium, with the slopes of the lines equaling the experimental values of K_app_ for these substrates in stopped-flow experiments.

The four remaining rate constants (*k*_3_-*k*_6_) are well-defined by global analysis. The pK_eq_ value calculated from the deprotonation/reprotonation rate constant ratio for the L-Ala external aldimine intermediate (2,600/255,000 s^−1^) is 2.0. The pK_a_ of the active site catalyst was determined from pH dependence studies to be < 6 (Zhou and Toney, 1999). Here, it is assumed to be 6. Combined, these values allow calculation of an L-Ala external aldimine C-H pK_a_ of 8.0. The C4′-H pK_eq_ is similarly calculated from the deprotonation/reprotonation rate constant ratio for the pyruvate ketimine intermediate (77/2,400 s^−1^) to be 1.5, corresponding to a C4′-H pK_a_ of 7.5.

For enzymatic deprotonation of the L-Ala external aldimine, ΔG^‡^ = 12.7 kcal/mol, while ΔG_0_ = 2.7 kcal/mol for proton transfer. These values give Δ${\text{G}}_{0}^{\text{‡}}$ = 11.3 kcal/mol (Table 2).

**Additional enzymes** Details of the calculations of the C-H pK_a_ values and intrinsic barriers for the other enzymes reported in Tables 1, 2 are provided in the Supplementary Material.

### Active Site C-H Acidity

The decrease in substrate C-H pK_a_ going from water to enzyme active site (i.e., ΔpK_a_ in Table 1) varies from ~7 for ketosteroid isomerase to ~23 for the PLP dependent enzymes tryptophan indole-lyase and tyrosine phenol-lyase. The average value for the cofactor independent enzymes is 12 ± 6 (using mean values of ranges) which corresponds to ~16 kcal/mol of carbanion stabilization by the enzymes, while that for the PLP dependent enzymes is 21 ± 2 which corresponds to ~29 kcal/mol of carbanion stabilization by the enzymes.

Richard et al. have shown that, in water, the pyridoxal protonation state used by enzymes lowers the C_α_-H pK_a_ of amino acids from ~29 to ~17 (Toth and Richard, 2007; Richard et al., 2009). Adjusting for this factor, the protein components of PLP enzymes reduce the C_α_-H pK_a_ of amino acids by an average of 9 ± 1 units, which is similar to the value for the cofactor independent enzymes. Thus, PLP itself provides the lion's share of carbanion stabilization in PLP dependent enzymes. The protein components provide ~12 kcal/mol of carbanion stabilization. In terms of potential active site interactions, this translates into ~6 hydrogen bonds, or ~3 salt bridges, or a combination thereof, that selectively stabilize the carbanion product over the reactant.

The calculated pK_a_ of the C_α_-H bond in the active site of aspartate aminotransferase is ~7. Bronsted analysis of the aspartate external aldimine intermediate in aspartate aminotransferase gave a β value of 0.62, and demonstrated strong steric hindrance toward exogenous catalysts, as expected for a reactant sequestered from solvent (Toney and Kirsch, 1989, 1992). Based on this Bronsted analysis, one can calculate a second-order rate constant for deprotonation of the active site-bound substrate by water and compare it to an experimentally estimated value for a resonance-stabilized carbon acid with pK_a_ = 7.

The second order rate constant for external aldimine deprotonation by water calculated from the previously reported analysis (log k_B_ = 0.62 × pK_a_−0.047 × molecular volume −2.1) when steric hindrance by the active site is eliminated (by assuming molecular volume = 0) is 7 × 10^−4^ M^−1^s^−1^. The experimentally derived rate constant for deprotonation of a resonance-stabilized C-H with pK_a_ = 7 is ~0.02 s^−1^, or ~4 × 10^−4^ M^−1^s^−1^ accounting for the concentration of water (Pearson and Dillon, 1953). The agreement between these independently derived values of the C-H deprotonation rate constants corroborates at least the aspartate aminotransferase pK_a_ reported in Table 1. In terms of Marcus theory, the similarity in the rate constants suggests that aspartate aminotransferase does little to reduce the intrinsic kinetic barrier to deprotonation compared to that in water, which is discussed further below.

QM/MM studies have been performed on several of the enzymes discussed here, providing theoretical estimates of C-H acidity in active sites through calculated FEPs. A study on ketosteroid isomerase gave a value of pK_eq_ that is essentially identical to the value calculated from the global optimization FEP reported here, providing excellent C-H pK_a_ agreement between theory and experiment (van der Kamp et al., 2013).

Proline racemase and similar cofactor-independent, two-cysteine amino acid racemases have been examined computationally (Stenta et al., 2008, 2009; Puig et al., 2009; Rubinstein and Major, 2009). The general conclusion from the computational studies is that no *stable* carbanionic intermediate exists, but that the reaction is a highly asynchronous, double proton transfer with the transition state essentially a fleeting carbanion. Based on experimental data, Albery and Knowles argued that a carbanionic intermediate does exists, although barely (Albery and Knowled, 1986). From molecular orbital considerations, electrophilic substitution reactions preferentially occur by front-side attack (Cram et al., 1955; Jensen and Gale, 1960; Sayre and Jensen, 1979). Therefore, it is reasonable to conclude that back-side double proton transfer in the proline racemase reaction effectively occurs through a carbanion, either a very short-lived intermediate or a transition state. The pK_a_ of this carbanionic species is calculated to be 15.8 from a DFT treatment and 21.6 from a semi-empirical one (Stenta et al., 2008). The former value agrees well with the experimental value reported in Table 1.

QM/MM calculations on the triosephophate isomerase reactions have produced a variety of energetic profiles, from which C-H pK_a_ values from ~14 to 20 for dihydroxyacetone phosphate, and a C-H pK_a_ value of 11 for glyceraldehyde phosphate, are calculated (Cui and Karplus, 2002; Guallar et al., 2004; Wang et al., 2006; Xiang and Warshel, 2008). The range for dihydroxyacetone phosphate is in general agreement with the experimental upper limit presented in Table 1, calculated from a refined experimental FEP (Toney, 2013). Mandelate racemase showed a shallow well in QM/MM studies for the carbanionic intermediate at 14 kcal/mol (Prat-Resina et al., 2005). This translates into a C-H pK_a_ value of 17, close to the upper limit of the experimental range. Finally, QM/MM calculations on alanine racemase yield a C-H pK_a_ value (12) that is in good agreement with experiment (11) (Major and Gao, 2006; Major et al., 2006). In general, QM/MM studies appear to provide accurate values for proton transfer equilibrium constants, and thereby accurate active site C-H pKa values.

The rates of proton transfers between heteroatoms such as nitrogen and oxygen are fast. For simple weak acids such as amines, carboxylic acids, alcohols, and water, proton association with the conjugate base is generally diffusion limited (10^10^-10^11^ M^−1^s^−1^). Acidity is determined by the wide variation in rate constants for proton dissociation from the acid form. For example, the rate constants for proton dissociation from acetic acid (pK_a_ = 4.8) in water is 7.8 × 10^5^ s^−1^, while that for *p*-nitrophenol (pK_a_ = 7.1) is 2.6 × 10^3^ s^−1^ (Isaacs, 1995).

The ionization of carbon acids is more complex. For example, a carbon acid with a pK_a_ similar to acetic acid (~5) dissociates a proton with a rate constant of ~1 s^−1^ in water (Pearson and Dillon, 1953). The large difference in the rates of ionization of heteroatoms vs. carbon exists because carbanions generally must be resonance stabilized to lower their pK_a_s to those of heteroatom-based acids. Scheme 1 shows the extensive resonance that occurs with PLP, where the carbanionic intermediate is stabilized via the azaallylic group as well as the pyridine ring.

The kinetic consequences of increasing carbon acidity by resonance delocalization have been elaborated by Bernasconi and given the name the “Principle of Non-perfect Synchronization” (Bernasconi, 1987, 1992, 2010). This principle can be summarized by noting that full resonance stabilization, which occurs only in the product and accounts for low pK_a_ values (i.e., thermodynamic stability of carbanions), requires full *p* orbital character at the reacting carbon. Conversely, transition states necessarily have only partial *p* orbital character, and are therefore only partially resonance stabilized compared to the product.

Marcus theory casts activation free energy (ΔG^‡^) in terms of the thermodynamic driving force of the reaction (ΔG_0_), and the intrinsic reaction barrier (Δ${\text{G}}_{0}^{\text{‡}}$; activation free energy for reaction when ΔG_0_ = 0) (Kresge and Silverman, 1999; Silverman, 2000). An excellent discussion of Marcus theory applied to enzymes is presented by Bearne and Spiteri (2005). In terms of Marcus theory, the intrinsic barrier to proton transfer is greater for carbon acids compared to heteroatom acids because of the late development of resonance stabilization with carbon.

Table 2 presents the intrinsic barriers to enzymatic proton transfers for which well-defined non-enzymatic and enzymatic values can both be calculated (see Supplementary Material). One fundamental catalytic mechanism that enzymes employ is selective stabilization (binding) of an intermediate, thereby lowering ΔG_0_ (Albery and Knowles, 1976). A second, equally important mechanism for catalysis is selective stabilization of transition states (catalysis of an individual step) (Albery and Knowles, 1976), thereby lowering Δ${\text{G}}_{0}^{\text{‡}}$. Within this context, the values in Table 2 show distinct behaviors for cofactor independent and PLP dependent enzymes.

Compared to non-enzymatic reactions, the two cofactor independent enzymes *decrease* intrinsic barriers to proton transfer by ~5 kcal/mol, while the PLP dependent enzymes *increase* intrinsic barriers by 2.4 ± 1.7 kcal/mol. The cofactor independent enzymes enhance the rate of proton transfer by stabilizing both the carbanion product (as evidenced by decreases in substrate C-H pK_a_s in active sites; Table 1) and the transition state leading to it (as evidenced by decreases in Δ${\text{G}}_{0}^{\text{‡}}$; Table 2). On the other hand, the PLP dependent enzymes presented in Table 2 (all of which employ PLP in the pyridine N-protonated form) achieve high rates of deprotonation exclusively by selective stabilization of the carbanionic intermediate (C-H pK_a_ reduction). Indeed, the high degree of carbanion stabilization on PLP enzymes is likely achieved by augmenting resonance stabilization through active site interactions with the cofactor, which inevitably leads to increased intrinsic barriers seen in Table 2.

Gerlt et al. (1991), Gerlt and Gassman (1992, 1993a,b), previously addressed a conundrum posed by carbon acid deprotonation in enzyme active sites: the large difference in pK_a_s of active site acid/base residues and substrate C-H makes deprotonation unfavorable. Central to their analysis is the idea that the intrinsic barriers to C-H deprotonation in active sites are similar to those in water (Gerlt and Gassman, 1992). If this were the case, then C-H pK_a_s would have to be reduced to that of the active site acid/base in order to account for the observed rates of enzymatic deprotonation. The authors championed concerted acid-base catalysis leading to enol intermediates in deprotonation of α-carbonyl compounds to account for the drastically reduced pK_a_s of substrates. The present analysis shows that enzymes can indeed lower intrinsic barriers to C-H deprotonation compared to reactions in water. This reduction in intrinsic barrier can be as large as ~7 kcal/mol in the case of proline racemase (Table 2), corresponding to a rate enhancement of ~10^5^ fold. For proline racemase, the experimental and computational evidence points to the transition state being a fleeting carbanion (Albery and Knowled, 1986; Stenta et al., 2008). As discussed in the Supplementary Material, this corresponds to a C-H pK_a_ of 16 in the active site (Table 1) and a difference in pK_a_ of ~9 units between the active site cysteine acid/base catalyst and the substrate. The enzyme achieves a high rate of C-H deprotonation not simply by lowering the C-H pK_a_ to that of the acid/base catalyst, but by coordinately lowering *both* ΔG_0_ and Δ${\text{G}}_{0}^{\text{‡}}$, as has been discussed previously (Bearne and Spiteri, 2005). For proline racemase, ΔΔG_rxn_ is ~-18 kcal/mol while ΔΔ${\text{G}}_{0}^{\text{‡}}$ ~-7 kcal/mol.

In conclusion, FEPs based on experimental data allow the calculation of substrate C-H pK_a_s in enzyme active sites based on the relation between pK_eq_ for the proton transfer and known pK_a_s of catalytic active site residues. The decreases in C-H pK_a_ provided by active sites ranges from moderate (~7 units) for relatively reactive substrates such as ketosteroids and triosephosphates to large (~20) for amino acids in PLP enzyme active sites. Calculations of Marcus intrinsic barriers for several reactions show that enzymes alter both intrinsic reaction barriers (catalysis of an individual step) and carbanion stability (selective binding of an intermediate) to achieve their impressive rate enhancements. The results presented here are an important step toward a complete quantitative understanding of the fundamental origins of enzyme catalysis.

## Author Contributions

The author confirms being the sole contributor of this work and has approved it for publication.

### Conflict of Interest Statement

The author declares that the research was conducted in the absence of any commercial or financial relationships that could be construed as a potential conflict of interest.

## Supplementary Material

Supplementary Material includes details of calculations of FEPs, C-H pK_a_s, and intrinsic barriers for the enzymes discussed (Data Sheet 1), as well as the COPASI input files used for global optimizations.
