# Supplementary material for: Carbon Acidity in Enzyme Active Sites
Source: Front Bioeng Biotechnol. 2019 Feb 19;7:25. doi: 10.3389/fbioe.2019.00025 (PMC6389717; doi:10.3389/fbioe.2019.00025)
Supplement: Supplementary file 1 [file Table_1.DOCX]

Supporting Information

Carbon Acidity in Enzyme Active Sites

Michael D. Toney

Department of Chemistry, University of California, Davis, CA 95616

**Cofactor Independent Enzymes**

**1) Ketosteroid isomerase (3-Oxo-Δ5-steroid isomerase)**


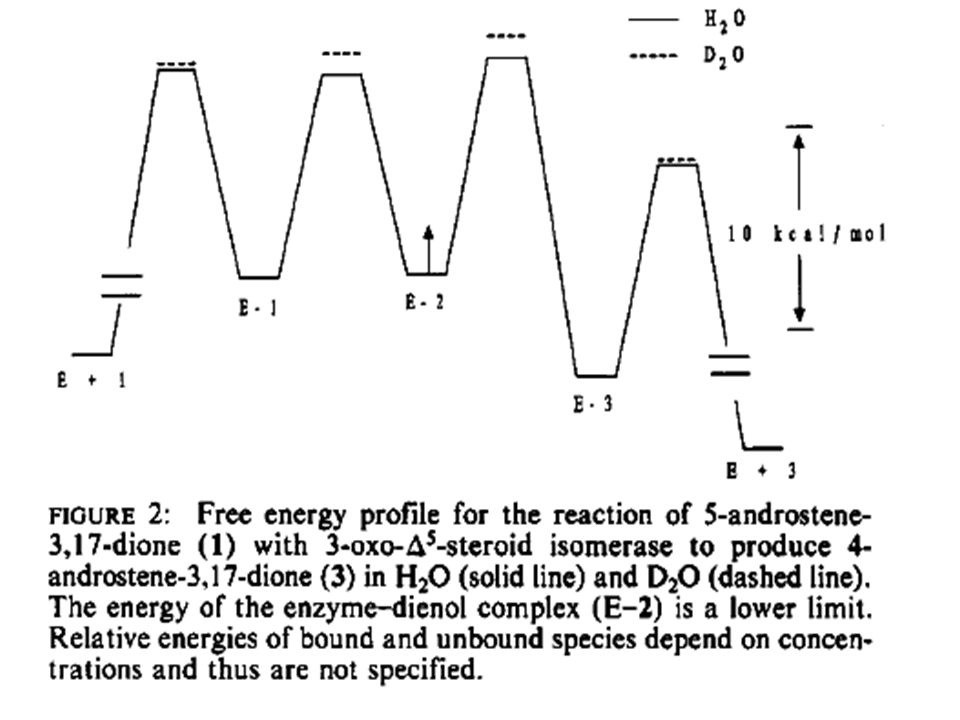
Pollack et al. studied the reaction catalyzed by ketosteroid isomerase (KSI) extensively. Their work, reproduced below, resulted in an nearly complete FEP calculated from a variety of different experiments.[*^1^*](#_ENREF_1)

**2) Proline racemase**

Knowles et al. performed extensive kinetic studies on proline racemase (ProR) that resulted in the construction of a FEP.[*^2-8^*](#_ENREF_2) Their results are reproduced below.

They addressed the question of the existence of a carbanionic intermediate and concluded that its existence is fleeting. Similarly, independent QM/MM studies on ProR draw the same conclusion, that the reaction occurs through a very carbanion-like transition state with little bond order to either of the two Cys catalytic acid/base residues.[*^9^*](#_ENREF_9)*^,^* [*^10^*](#_ENREF_10) No distinct carbanionic intermediate was located. The conclusion that the transition state is essentially a carbanion whose existence is too short (less than a few molecular vibrations) to define it as an intermediate does not preclude the present analysis: here, the free energy of activation is taken to be the free energy of the proton transfer equilibrium. Given the preference, due to molecular orbital interactions, of S_E_2 protonolysis reactions for front-side attack, it is not unexpected for the ProR reaction to have a fully carbanionic transition state since the active site geometry enforces back-side attack to achieve stereoinversion.[*^11-13^*](#_ENREF_11)

Based on the above reasoning, the reported rate constant for deprotonation (5200 s^-1^) was used to calculate a free energy of activation of 12.3 kcal/mol. *This value is also assumed to be the free energy of proton transfer between the C_α_-H of proline and the cysteine thiolate in the active site (pK_eq_).* The value of pK_eq_, for a proton transfer reaction that is 12.3 kcal/mol endergonic, is 9.0. Combining this value with that of the active site cysteines (~7)[*^14^*](#_ENREF_14) yields an active site C-H pK_a_ value of 16. The pK_a_ of proline in solution is 29.[*^15^*](#_ENREF_15)

For the enzymatic reaction ΔG^‡^_int_ = 3.2 kcal/mol based on the above values. If the carbanion is assumed to be 1 kcal/mol more stable than the transition state for its formation, then k_reprot_ = 1× 10^12^ s^-1^ and ΔG^‡^_int_ = 5.1 kcal/mol. This value is reported in Table 2. The ΔG^‡^_int_ for the nonenzymatic reaction was estimated using the value of k_HO-_ = 4.5 × 10^-5^ M^-1^s^-1^ reported by Williams *et al.* [*^15^*](#_ENREF_15) This second order rate constant was converted to a first order rate constant for deprotonation in the encounter complex using the base/C-H association constant of 0.017 M^-1^ estimated by Hine and commonly employed in the literature.[*^16^*](#_ENREF_16) This leads to a value of 2.6 × 10^-3^ s^-1^, which corresponds to ΔG^‡^ = 21 kcal/mol. ΔG_rxn_ = 17.7 kcal/mol is calculated from the difference in pK_a_ values of water and the C-H of proline. These values can be used to calculate ΔG^‡^_int_ = 10.5 kcal/mol for the reaction with hydroxide in solution.

**3) Triosephosphate isomerase** Knowles et al. reported the FEP for triosphosphate isomerase (TIM).[*^17-26^*](#_ENREF_17) They were unable to define the energy of the central, carbanionic enediolate intermediate. Previous work by this author reexamined the TIM FEP using global optimization.[*^27^*](#_ENREF_27) The results of that study are reproduced below.
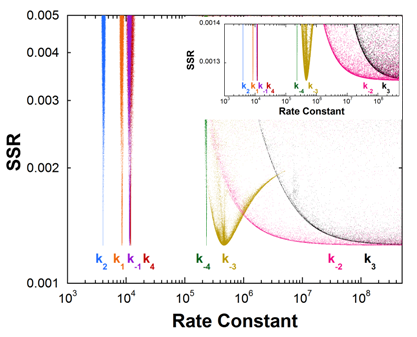


The rate constant for deprotonation of DHAP in the active site was found to be 4000 s^-1^, while the range of the rate constant for reprotonation of the enolate to give DHAP is between 10^8^ and 10^12^ s^-1^. We assume the latter to be the theoretical limit at 25 °C. The proton transfer pK_eq_ calculated from these rate constants is between 4.4 and 8.4. Combined with the pK_a_ of 6 for the Glu165 active site acid/base catalyst, a range of C-H pK_a_ values from 10.4 to 14.4 is calculated. This range is reported as 10-14 in Table 1. Similarly, the rate constant for deprotonation of GAP in the active site is 500,000 s^-1^ while the range for the reprotonation rate constant is 10^9^ – 10^12^ s^-1^. This translates into a C-H pK_a_ range of 9.3-12.3, which reported at 9-12 in Table 1. The pK_a_ for DHAP in solution is 18.[*^11^*](#_ENREF_11) The pK_a_ for GAP is taken as that for acetaldehyde (17).

The intrinsic barrier for TIM catalyzed DHAP deprotonation can be calculated as a range of values from the range of pK_eq_ = 4.4 – 8.4 and the ΔG^‡^ = 12.5 kcal/mol. The range of ΔG^‡^_int_ is 5.3 – 9.3 kcal/mol. The intrinsic barrier for the nonenzymatic deprotonation of DHAP was calculated using acetone as a model. The pK_a_ of acetone in water at 25 °C is 19.2 and the rate constant for hydroxide catalyzed deprotonation is 0.173 M^-1^s^-1^.[*^28^*](#_ENREF_28) The calculated rate constant for deprotonation in the hydroxide/acetone encounter complex (assuming an association constant of 0.017 M^-1^ [*^16^*](#_ENREF_16)) is 0.173 M^-1^s^-1^/0.017 M^-1^ = 10.2 s^-1^, corresponding to ΔG^‡^ = 16.0 kcal/mol. ΔG_rxn_ for this proton transfer is 4.7 kcal/mol. These values give ΔG^‡^_int_ = 13.5 kcal/mol.

**Pyridoxal Phosphate Dependent Enzymes**

Calculation of the equilibrium constant for formation of the carbanionic quinonoid intermediate for all PLP enzymes used an extinction coefficient of 8,000 M^-1^ cm^-1^ for external aldimine intermediates[*^29^*](#_ENREF_29) and 40,000 M^-1^ cm^-1^ for quinonoid intermediates.[*^30^*](#_ENREF_30)*^,^* [*^31^*](#_ENREF_31) These values are expected to vary slightly between enzymes but not more than 2-fold.

**4) Tryptophan synthase**

Phillips et al. studied the formation of the relatively stable quinonoid intermediate formed on reaction of L-tryptophan with tryptophan synthase.[*^32^*](#_ENREF_32) Their data can be used to calculated an equilibrium constant of 0.21 for formation of the carbanionic quinonoid intermediate on the enzyme. The pK_eq_ of 0.21 combined with the active site acid/base pK_a_ of 7.3[*^33^*](#_ENREF_33) gives C-H pK_a_ of 8.0 for the external aldimine intermediate in the active site.

Deprotonation of the L-tryptophan external aldimine intermediate occurs with a rate constant of ~20 s^-1^.[*^34^*](#_ENREF_34)*^,^* [*^35^*](#_ENREF_35) This corresponds to ΔG^‡^ = 15.6 kcal/mol. Based on pK_eq_ = 0.21, ΔG_rxn_ = 0.29 kcal/mol. These values correspond to ΔG^‡^_int_ = 15.4 kcal/mol. Tryptophan synthase binds the PLP cofactor in the pyridine nitrogen-unprotonated form.[*^36^*](#_ENREF_36) Nonenzymatic reactions of Gly-pyridoxal aldimines in this protonation state are not detectable.[*^37^*](#_ENREF_37) Therefore, an intrinsic barrier for the equivalent reaction in solution cannot be calculated.

**5) Tryptophan indole-lyase**

The reaction of tryptophan indole-lyase with tryptophan was studied by stopped-flow kinetics, and a prominent absorption band for the quinonoid intermediate was observed.[*^38^*](#_ENREF_38) The pK_eq_ for external aldimine deprotonation calculated from the spectral data is 0.7. The pH dependence of k_cat_ for this enzyme is flat between pH 6 and 9. The active site lysine must be deprotonated to act as a catalytic base; hence, the pK_a_ of this group must be less than 6. Here, it is taken to be ~5. The active site C-H pK_a_ is therefore ~5.7, reported as 6 in Table 1.

The enzymatic rate constant for deprotonation of the L-tryptophan external aldimine is 940 s^-1^.[*^39^*](#_ENREF_39) This corresponds to ΔG^‡^ = 13.3 kcal/mol, while ΔG_rxn_ = 0.95 kcal/mol. These values give ΔG^‡^_int_ = 12.8 kcal/mol. Here, the observed ΔG^‡^ is very close to ΔG^‡^_int_ since the equilibrium constant for the proton transfer at the active site (K_eq_ = 0.2) is close to unity.

The hydroxide catalyzed nonenzymatic reaction of the Gly-pyridoxal aldimine has a rate constant of 750 M^-1^s^-1^ for the N-protonated form employed by the enzyme.[*^37^*](#_ENREF_37) Correcting with the assumed association constant of 0.017 M^-1^ for hydroxide forming the productive reaction complex gives a first order rate constant of 4.4 × 10^4^ s^-1^, and ΔG^‡^ = 11.0 kcal/mol. The C-H pK_a_ for the aldimine in solution is 17 [*^37^*](#_ENREF_37) and ΔG_rxn_ for proton transfer is 1.3 kcal/mol. These values correspond to ΔG^‡^_int_ = 10.4 kcal/mol. For comparison, 4-phenacylpyridinium carbon acids studied by Stephanidis and Bunting show intrinsic barriers of 14 – 15 kcal/mol.[*^40^*](#_ENREF_40) These model compounds lack the 3’-phenolate group of the PLP protonation state employed by tryptophan indole-lyase, as well as the electrostatic stabilization by the iminium ion, both of which are expected to reduce resonance stabilization of the carbanion and thereby the intrinsic barrier. We have therefore chosen to report in Table 2 the value of 10.4 calculated above for the Gly-pyridoxal aldimine. *This value is used for all of the PLP enzymes listed in Table 2.*

**6) Tyrosine phenol-lyase**

Quinonoid formation with tyrosine phenol-lyase was studied in the context of the D214A and D214N mutations and their effects on quinonoid stability.[*^41^*](#_ENREF_41) Asp214 interacts with the protonated pyridine nitrogen of PLP in this enzyme. The D214A and D214N mutants show no detectable quinonoid formation with L-phenylalanine (which does not react further than the quinonoid), while wild type enzyme does. The pK_eq_ calculated from the quinonoid absorbance of the latter is 0.2. The value of k_cat_ is independent of pH for the reaction of tyrosine with the wild type enzyme between 6.5 and 9.5.[*^42^*](#_ENREF_42) The pK_a_ of the catalytic lysine in the external aldimine intermediate is assumed to be 6 based on this result. These values combine to give a C-H pK_a_ of 6.2 for tyrosine in the active site, reported as 6 in Table 1.

The rate constant for deprotonation of the phenylalanine external aldimine in the enzyme active site is 16 s^-1^, or ΔG^‡^ = 15.7 kcal/mol. The calculated K_eq_ gives ΔG_rxn_ = 0.27 kcal/mol for proton transfer. These values correspond to ΔG^‡^_int_ = 15.5 kcal/mol. Here, the observed ΔG^‡^ is very close to ΔG^‡^_int_ since the equilibrium constant for the proton transfer at the active site (K_eq_ = 0.6) is close to unity.

**7) Alanine racemase**


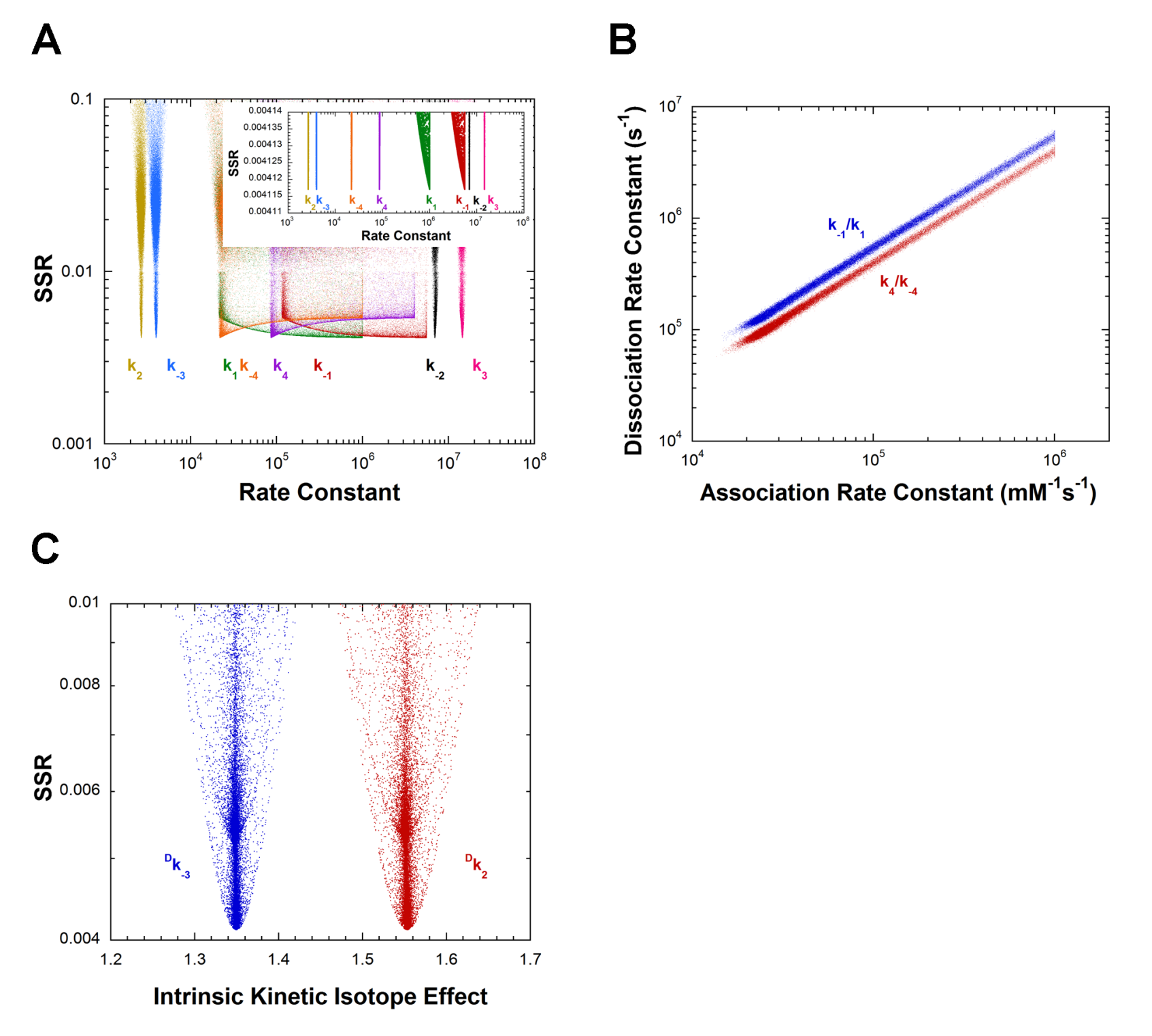
The FEP for alanine racemase was determined previously by global optimization.[*^27^*](#_ENREF_27) The results are reproduced below. The ratios of the rate constants for deprotonation and reprotonation of both external aldimine stereosiomers were used to calculate pK_eq_ values for formation of the carbanionic quinonoid intermediate. These values are 3.4 for the L isomer and 3.5 for the D isomer. The absorbance of the quinonoid intermediate was also used to calculate a similar value of 3.7. The pK_a_ of the active site acid/base catalyst in the external aldimine intermediate is 7.4 [*^43^*](#_ENREF_43) leading to an external aldimine C-H pK_a_ of 10.8-11.1, which is reported as 11 in Table 1.

The rate constant for deprotonation of the L-alanine external aldimine intermediate on the enzyme is 2600 s^-1^ [*^27^*](#_ENREF_27) and ΔG^‡^ = 12.7 kcal/mol. ΔG_rxn_ = 4.6 kcal/mol for proton transfer. These values give ΔG^‡^_int_ = 10.3 kcal/mol. The ΔG^‡^_int_ value for the nonenzymatic reaction is not available for the pyridine nitrogen-unprotonated form of the aldimine in water, as discussed above for tryptophan synthase.

**References**

[1] Hawkinson, D. C., Eames, T. C., and Pollack, R. M. (1991) Energetics of 3-oxo-delta 5-steroid isomerase: source of the catalytic power of the enzyme, *Biochemistry* *30*, 10849-10858.

[2] Fisher, L. M., Albery, W. J., and Knowles, J. R. (1986) Energetics of proline racemase: racemization of unlabeled proline in the unsaturated, saturated, and oversaturated regimes, *Biochemistry* *25*, 2529-2537.

[3] Belasco, J. G., Bruice, T. W., Fisher, L. M., Albery, W. J., and Knowles, J. R. (1986) Energetics of proline racemase: rates, fractionation factors, and buffer catalysis in the oversaturated region. Nature of the interconversion of the two forms of free enzyme, *Biochemistry* *25*, 2564-2571.

[4] Belasco, J. G., Bruice, T. W., Albery, W. J., and Knowles, J. R. (1986) Energetics of proline racemase: fractionation factors for the essential catalytic groups in the enzyme-substrate complexes, *Biochemistry* *25*, 2558-2564.

[5] Belasco, J. G., Albery, W. J., and Knowles, J. R. (1986) Energetics of proline racemase: double fractionation experiment, a test for concertedness and for transition-state dominance, *Biochemistry* *25*, 2552-2558.

[6] Fisher, L. M., Belasco, J. G., Bruice, T. W., Albery, W. J., and Knowles, J. R. (1986) Energetics of proline racemase: transition-state fractionation factors for the two protons involved in the catalytic steps, *Biochemistry* *25*, 2543-2551.

[7] Fisher, L. M., Albery, W. J., and Knowles, J. R. (1986) Energetics of proline racemase: tracer perturbation experiments using [14C]proline that measure the interconversion rate of the two forms of free enzyme, *Biochemistry* *25*, 2538-2542.

[8] Albery, W. J., and Knowles, J. R. (1986) Energetics and Mechanism of Proline Racemase, *Biochemistry* *25*, 2572-2577.

[9] Rubinstein, A., and Major, D. T. (2009) Catalyzing racemizations in the absence of a cofactor: the reaction mechanism in proline racemase, *J Am Chem Soc* *131*, 8513-8521.

[10] Stenta, M., Calvaresi, M., Altoe, P., Spinelli, D., Garavelli, M., and Bottoni, A. (2008) The catalytic activity of proline racemase: a quantum mechanical/molecular mechanical study, *J Phys Chem B* *112*, 1057-1059.

[11] Richard, J. P. (1984) Acid-Base Catalysis of the Elimination and Isomerization-Reactions of Triose Phosphates, *J Am Chem Soc* *106*, 4926-4936.

[12] Isaacs, N. S. (1995) *Physical organic chemistry*, 2nd ed., Longman Scientific & Technical ;

Wiley & Sons, Burnt Mill, Harlow, Essex, England

New York, N.Y.

[13] Fukuto, J. M., and Jensen, F. R. (1983) Mechanisms of Se2 Reactions - Emphasis on Organotin Compounds, *Accounts Chem Res* *16*, 177-184.

[14] Cardinale, G. J., and Abeles, R. H. (1968) Purification and mechanism of action of proline racemase, *Biochemistry* *7*, 3970-3978.

[15] Williams, G., Maziarz, E. P., 3rd, Amyes, T. L., Wood, T. D., and Richard, J. P. (2003) Formation and stability of the enolates of N-protonated proline methyl ester and proline zwitterion in aqueous solution: a nonenzymatic model for the first step in the racemization of proline catalyzed by proline racemase, *Biochemistry* *42*, 8354-8361.

[16] Hine, J. (1971) Rate and equilibrium in the addition of bases to electrophilic carbon and in SN1 reactions, *J Am Chem Soc* *93*, 3701-3708.

[17] Nickbarg, E. B., and Knowles, J. R. (1988) Triosephosphate isomerase: energetics of the reaction catalyzed by the yeast enzyme expressed in Escherichia coli, *Biochemistry* *27*, 5939-5947.

[18] Blacklow, S. C., Raines, R. T., Lim, W. A., Zamore, P. D., and Knowles, J. R. (1988) Triosephosphate isomerase catalysis is diffusion controlled. Appendix: Analysis of triose phosphate equilibria in aqueous solution by 31P NMR, *Biochemistry* *27*, 1158-1167.

[19] Belasco, J. G., Herlihy, J. M., and Knowles, J. R. (1978) Critical ionization states in the reaction catalyzed by triosephosphate isomerase, *Biochemistry* *17*, 2971-2978.

[20] Albery, W. J., and Knowles, J. R. (1976) Free-energy profile of the reaction catalyzed by triosephosphate isomerase, *Biochemistry* *15*, 5627-5631.

[21] Fisher, L. M., Albery, W. J., and Knowles, J. R. (1976) Energetics of triosephosphate isomerase: the nature of the proton transfer between the catalytic base and solvent water, *Biochemistry* *15*, 5621-5626.

[22] Leadlay, P. F., Albery, W. J., and Knowles, J. R. (1976) Energetics of triosephosphate isomerase: deuterium isotope effects in the enzyme-catalyzed reaction, *Biochemistry* *15*, 5617-5620.

[23] Fletcher, S. J., Herlihy, J. M., Albery, W. J., and Knowles, J. R. (1976) Energetics of triosephosphate isomerase: the appearance of solvent tritium in substrate glyceraldehyde 3-phosphate and in product, *Biochemistry* *15*, 5612-5617.

[24] Maister, S. G., Pett, C. P., Albery, W. J., and Knowles, J. R. (1976) Energetics of triosephosphate isomerase: the appearance of solvent tritium in substrate dihydroxyacetone phosphate and in product, *Biochemistry* *15*, 5607-5612.

[25] Herlihy, J. M., Maister, S. G., Albery, W. J., and Knowles, J. R. (1976) Energetics of triosephosphate isomerase: the fate of the 1(R)-3H label of tritiated dihydroxyacetone phsophate in the isomerase reaction, *Biochemistry* *15*, 5601-5607.

[26] Knowles, J. R., Leadlay, P. F., and Maister, S. G. (1972) Triosephosphate isomerase: isotope studies on the mechanistic pathway, *Cold Spring Harb Symp Quant Biol* *36*, 157-164.

[27] Toney, M. D. (2013) Common enzymological experiments allow free energy profile determination, *Biochemistry* *52*, 5952-5965.

[28] Guthrie, J. P. (1991) Rate Equilibrium Correlations for the Aldol Condensation - an Analysis in Terms of Marcus Theory, *J Am Chem Soc* *113*, 7249-7255.

[29] Goldberg, J. M., Swanson, R. V., Goodman, H. S., and Kirsch, J. F. (1991) The tyrosine-225 to phenylalanine mutation of Escherichia coli aspartate aminotransferase results in an alkaline transition in the spectrophotometric and kinetic pKa values and reduced values of both kcat and Km, *Biochemistry* *30*, 305-312.

[30] Metzler, C. M., Harris, A. G., and Metzler, D. E. (1988) Spectroscopic studies of quinonoid species from pyridoxal 5'-phosphate, *Biochemistry* *27*, 4923-4933.

[31] Mozzarelli, A., Peracchi, A., Rovegno, B., Dale, G., Rossi, G. L., and Dunn, M. F. (2000) Effect of pH and monovalent cations on the formation of quinonoid intermediates of the tryptophan synthase alpha(2)beta(2) complex in solution and in the crystal, *J Biol Chem* *275*, 6956-6962.

[32] Phillips, R. S., McPhie, P., Miles, E. W., Marchal, S., and Lange, R. (2008) Quantitative effects of allosteric ligands and mutations on conformational equilibria in Salmonella typhimurium tryptophan synthase, *Arch Biochem Biophys* *470*, 8-19.

[33] Ro, H. S., and Wilson Miles, E. (1999) Catalytic mechanism of the tryptophan synthase alpha(2)beta(2) complex. Effects of pH, isotopic substitution, and allosteric ligands, *J Biol Chem* *274*, 31189-31194.

[34] Cash, M. T., Miles, E. W., and Phillips, R. S. (2004) The reaction of indole with the aminoacrylate intermediate of Salmonella typhimurium tryptophan synthase: observation of a primary kinetic isotope effect with 3-[(2)H]indole, *Arch Biochem Biophys* *432*, 233-243.

[35] Drewe, W. F., Jr., Koerber, S. C., and Dunn, M. F. (1989) Application of rapid-scanning, stopped-flow spectroscopy to the characterization of intermediates formed in the reactions of L- and D-tryptophan and beta-mercaptoethanol with Escherichia coli tryptophan synthase, *Biochimie* *71*, 509-519.

[36] Caulkins, B. G., Young, R. P., Kudla, R. A., Yang, C., Bittbauer, T. J., Bastin, B., Hilario, E., Fan, L., Marsella, M. J., Dunn, M. F., and Mueller, L. J. (2016) NMR Crystallography of a Carbanionic Intermediate in Tryptophan Synthase: Chemical Structure, Tautomerization, and Reaction Specificity, *J Am Chem Soc*.

[37] Toth, K., and Richard, J. P. (2007) Covalent catalysis by pyridoxal: evaluation of the effect of the cofactor on the carbon acidity of glycine, *J Am Chem Soc* *129*, 3013-3021.

[38] Phillips, R. S. (1991) Reaction of indole and analogues with amino acid complexes of Escherichia coli tryptophan indole-lyase: detection of a new reaction intermediate by rapid-scanning stopped-flow spectrophotometry, *Biochemistry* *30*, 5927-5934.

[39] Phillips, R. S. (1989) Mechanism of Tryptophan Indole-Lyase - Insights from Pre-Steady-State Kinetics and Substrate and Solvent Isotope Effects, *J Am Chem Soc* *111*, 727-730.

[40] Stefanidis, D., and Bunting, J. W. (1991) Transition-State Imbalance in the General-Base Catalysis of the Deprotonation of 4-Phenacylpyridinium Cations, *J Am Chem Soc* *113*, 991-995.

[41] Demidkina, T. V., Faleev, N. G., Papisova, A. I., Bazhulina, N. P., Kulikova, V. V., Gollnick, P. D., and Phillips, R. S. (2006) Aspartic acid 214 in Citrobacter freundii tyrosine phenol-lyase ensures sufficient C--H-acidity of the external aldimine intermediate and proper orientation of the cofactor at the active site, *Biochim Biophys Acta* *1764*, 1268-1276.

[42] Faleev, N. G., Spirina, S. N., Ivoilov, V. S., Demidkina, T. V., and Phillips, R. S. (1996) The catalytic mechanism of tyrosine phenol-lyase from Erwinia herbicola: the effect of substrate structure on pH-dependence of kinetic parameters in the reactions with ring-substituted tyrosines, *Z Naturforsch C* *51*, 363-370.

[43] Sun, S., and Toney, M. D. (1999) Evidence for a two-base mechanism involving tyrosine-265 from arginine-219 mutants of alanine racemase, *Biochemistry* *38*, 4058-4065.
